# The relationship between acquaintance with a SARS-CoV-2 death, positive SARS-CoV-2 test results, and sleep duration among college students in fall 2020

**DOI:** 10.3389/fpubh.2022.949438

**Published:** 2022-08-18

**Authors:** Edlin Garcia Colato, Chen Chen, Sina Kianersi, Christina Ludema, Molly Rosenberg, Jonathan T. Macy

**Affiliations:** ^1^Department of Applied Health Science, Indiana University School of Public Health Bloomington, Bloomington, IN, United States; ^2^Department of Epidemiology and Biostatistics, Indiana University School of Public Health Bloomington, Bloomington, IN, United States

**Keywords:** sleep duration, SARS-CoV-2, COVID-19, college students, public health

## Abstract

**Background:**

The aim of this study was to test whether two SARS-CoV-2 experiences, knowing someone who had died of SARS-CoV-2 infection and having received a positive SARS-CoV-2 test result, were associated with shorter sleep duration among undergraduate students.

**Methods:**

An online cross-sectional study was conducted at a large public Midwestern university in September 2020 (fall semester). Self-reported average sleep duration and the exposures of interest, knowing someone who died from a SARS-CoV-2 infection and their own SARS-CoV-2 test result, were collected from 1,058 undergraduate study participants.

**Results:**

Respondents who knew someone who had died of a SARS-CoV-2 infection were more likely to report having a short sleep duration, compared to respondents who did not know someone who had died of a SARS-CoV-2 infection (aOR = 1.80, 95% CI: 1.14, 2.79). However, those with a positive SARS-CoV-2 test result were less likely to report a short sleep duration, compared to respondents without a positive test history (aOR = 0.47, 95% CI: 0.21, 0.91).

**Conclusions:**

These findings suggest that college students' knowing someone who had died of SARS-CoV-2 infection and having received a positive SARS-CoV-2 test result are associated with sleep duration. However, different experiences may impact sleep differently, so further research is warranted to better understand how unusual events impact the sleep of college students.

## Introduction

### Background and rationale

Sleep is associated with overall physical and mental health. Insufficient sleep weakens the immune system, making individuals more susceptible to infections, including contracting SARS-CoV-2, and, once infected, less able to mount a strong immune response (1). Sleep duration is one dimension used to measure sleep health (2, 3). Within the US, the National Sleep Foundation (NSF) as well as the American Academy of Sleep Medicine (AASM) alongside with the Sleep Research Society (SRS) have made sleep duration recommendations (2, 4). The NSF recommends between seven and 9 h for adults ages 18–64 (5). Insufficient/short sleep duration is sleeping less than the recommended minimum 7 h for adults and can negatively affect an individual's overall health. Inadequate sleep duration has been linked to diabetes, hypertension, cardiovascular diseases, obesity, depression, and anxiety (6–8).

For college students, poor sleep health is negatively associated with student academic performance and graduation (9, 10). Student living space, such as residing on campus in dormitories, can disrupt sleeping patterns (11, 12). This disruption in the environment is most likely to be experienced by first-year college students, who are usually required to sleep in dormitories, and for many it's their first time experiencing living away from home. Furthermore, during college, approximately 40% of enrolled college students balance their academic responsibilities with work demands (13). There is reason to believe that work-related factors might also affect student sleep duration (14). Increased hours at work, in addition to coursework, can result in less available time for sleep (9). This is likely worse for Black and Hispanic full-time undergraduate students, who have a higher proportion of working 35 or more hours a week, 18.5% and 13.6%, respectively, compared to their White counterparts (9.7%) (15).

Starting in 2020, the COVID-19 pandemic disrupted individual's personal, school, and work routines, including sleep, likely because of the stress associated with the pandemic (16). Short and long average sleep durations were found to be more prevalent than meeting the recommended sleep duration (17). Prior research has found that quarantining during COVID-19 negatively affected Jordanian university students' sleep quality and depressive symptoms (18). However, less is known about college students' sleep duration post-lockdown during fall 2020, their first semester back on campus, and whether there was an association with knowing someone who died from SARS-CoV-2 infection and having tested positive for SARS-CoV-2 and poor sleep duration.

One potential contributing factor to poor sleep among college students is having a personal experience with SARS-CoV-2. For example, individuals who have not been infected with SARS-CoV-2 may experience worry and fear of contagion of SARS-CoV-2. This can negatively impact their mental health, which has been documented in past epidemics (19), potentially affecting sleep. By the start of the fall 2020 semester, at the end of August 2020, there were over 25 million reported COVID-19 cases, and 800,000 reported COVID-19 deaths worldwide, cumulative from December 2019 (20). In the US alone, there were nearly 6 million cumulative cases and nearly 181,000 overall deaths (20). Individuals from past epidemics (e.g., Ebola) who have experienced significant loss have struggled with grief and coping long after their loved ones' passing (21). This type of personal experience could also possibly negatively impact sleep.

### Objectives

The aim of this study was to test whether two SARS-CoV-2 experiences, knowing someone who had died from a SARS-CoV-2 infection and having had a positive SARS-CoV-2 test result, were associated with short sleep duration among undergraduate college students during their first semester on campus since the beginning of the pandemic. We hypothesized that students with personal connections to SARS-CoV-2 infections and death would report shorter sleep durations. We used the Strengthening the Reporting of Observational Studies in Epidemiology (STROBE) for cross-sectional studies checklist.

## Methods

### Study design and setting

This study used data from the baseline survey of the “IU COVID Serosurvey” conducted in September 2020 at a large Midwestern public university with a Fall 2020 semester enrollment of approximately 32,000 undergraduate students (22). All data were collected through the Research Electronic Data Capture (REDCap) (23), a data management system (23, 24). The parent study was a randomized controlled trial focused on student behavior based on SARS-CoV-2 antibody status knowledge. Electronic written voluntary consent was also recorded in REDCap. Participants completed a 30-min baseline survey and received compensation for completing the questionnaire, with a maximum of $30 for completing all parent study activities. This study (Protocol #2008293852) was approved by Indiana University's Human Subjects and Institutional Review Board, and all participants provided informed consent. This current cross-sectional study does not use a per-protocol analysis.

### Participants

The Office of the Vice Provost for Undergraduate Education generated a random list of 7,499 undergraduates, representative of the undergraduate student population (~32,620). Eligible undergraduates were ages 18 or older, were residing in the same county as the university at the time of the study, and were actively enrolled at the university. In September 2020, study information was emailed to the randomly selected sample of undergraduates, inviting them to enroll in the study and including unique student links to access the online survey. A total of 1,397 (18.6%) out of 7,499 invited students consented to participate, of which 1,264 (90.5%) responded to the baseline survey at some capacity. Additional details on the baseline survey are described in a separate article (22).

The inclusion criteria used for this study included age, credit hours, and sleep duration. As per NSF's sleep recommendation by age, the sample for this study was restricted to ages 18 to 64 and *n* = 1 who were above age 64 was excluded. We also restricted the sample to those actively enrolled in one or more credit hours (*n* = 1 enrolled in 0 credits). The final inclusion criteria were sleep duration equal to or less than 9 h. Similar to Lu et al. (25), in which only 1.7% of their respondents reported averaging a long sleep duration, we also restricted the analyses to only compare short sleep duration to the recommended sleep duration. Twenty-three observations (1.8%) were excluded because they exceeded the 9-h cutoff and were considered long sleep duration, yielding *n* = 1,239.

Of the 1,239 eligible participants, the sample was further filtered by sequentially removing participants with missing data as follows: *n* = 76 for age, *n* = 1 for sex, *n* = 1 for gender, *n* = 10 for credit hours, *n* = 3 for year in college, *n* = 2 job for with in-person interaction, *n* = 1 for general health, *n* = 64 for depressive symptoms, *n* = 20 for stress symptoms, *n* = 3 for nicotine use, and *n* = 1 for alcohol use. Thus, the final sample for analyses was 1,058 students who met all eligibility criteria and had complete data. Compared to the respondents who were included in the study, more males, African American/Black participants, Asian participants, and students in their second year of college studies were excluded because of non-response (all *p*-values < 0.05).

### Variables

#### Outcome: Sleep duration

Sleep duration was captured using the question, “*On average, how many hours of sleep do you get a night?*” with accepted response options for the number of hours ranging from 0 to 24 h. Responses were recoded to 1 = “less than 7 h; short/insufficient sleep duration” and 0 = “seven to 9 h; appropriate/recommended sleep duration”, based on NSF's classification of sleep duration (5, 26).

#### Exposures: SARS-CoV-2 variables

**Knew Someone who Died From a SARS-CoV-2 Infection**. “*Have you known someone who has died of COVID-19?*” Response options were no, yes, and don't know. The responses for “don't know” were combined with all the “no” responses, yielding two groups: no/don't know vs. yes.

**Tested Positive for a SARS-CoV-2 Infection (Self)**. “*Have you ever tested positive for a SARS-CoV-2 (COVID-19) infection?”* Response options were no, yes, and don't know. Responses for “don't know” and “no” were combined, yielding two groups: no/don't know vs. yes. During the time of this study students were regularly tested by the university and home-based self-tests were not readily available to the general public. Therefore, the self-reported positive SARS-CoV-2 test results are considered to be PCR tests administered by either the university, a health care provider, or public health agency.

### Covariates

Covariates were age (continuous), sex (female vs. male) (25), race (African American/Black, Asian, more than one race, some other race, or White) (15), ethnicity (Hispanic vs. non-Hispanic/Latinx) (15), perceived general health (excellent, very good, good, fair/pair) (6–8), residence type (on-campus/dormitories vs. not on-campus/dormitories) (11, 12), year in college (first, second, third, fourth, fifth or more) (10), total credit hours (≤12 vs. >12), having a job with in-person interaction (no vs. yes) (14), past 30-day nicotine use (no vs. yes) (27), alcohol use (no vs. yes) (28), depressive symptoms (CES-D-10; <10 vs. ≥10) (29–31), and perceived stress symptoms (PSS-10; continuous) (32), Additional information about the sociodemographic variables and questions asked in the baseline survey can be found in the Appendix.

### Statistical methods

Pearson's chi-square tests and *t*-tests were conducted to examine the bivariate relationships between the selected covariates and sleep duration (low vs. recommended).

Logistic regression models examined whether each of the knowing someone who had died of SARS-CoV-2 infection and having received a positive SARS-CoV-2 test result were associated with short vs. recommended sleep duration. Both models adjusted for the following covariates: age, sex, race, ethnicity, general health, residence type, year in college, total credit hours, job with in-person interaction, nicotine use, alcohol use, depressive symptoms, and perceived stress symptoms. All data were analyzed with RStudio software (Version 1.4.1106).

## Results

### Participants and descriptive data

Table 1 summarizes the socio-demographic characteristics by sex. Table 2 summarizes the socio-demographic characteristics of the 1,058 students by sleep duration (short, appropriate/recommended). The mean age of the student participants was approximately 20.0 years (SD = 1.92). Most respondents were female (669; 64.3%), White (861; 81.4%), Non-Hispanic/Latinx (974; 92.1%), were enrolled in more than 12 total credit hours (935; 88.4%), lived off-campus (744; 70.3%), did not have a job that required in-person interaction (760; 71.8%), did not report depressive symptoms (616; 58.2%), reported moderate perceived stress symptoms (700; 66.2%), did not use nicotine products in the past 30-days (729; 68.9%), drank alcohol (718; 67.9%), did not know someone who died of a SARS-CoV-2 infection (924; 87.3%), and had never tested positive for a SARS-CoV-2 infection (947; 89.5%).

**Table 1:** Characteristics of undergraduates by sex.

**Characteristics**	**Total sample *n =* 1,058**	**Female *n =* 680**	**Male *n =* 378**	***p*-value**
Total %	100.0	64.3	35.7	
**Socio-demographic variables**			
**Age** (mean, sd)	19.96 (1.92)	19.89 (1.80)	20.08 (2.12)	0.14
**Sleep Duration** (*n*, %)				0.25
4–6 hours/day	179 (16.9)	118 (17.4)	61 (16.1)	
7–9 hours/day	879 (83.1)	562 (82.6)	317 (83.9)	
**Gender (** ***n*** **, %)**				
Female	669 (63.2)	669 (98.4)	0 (0)	
Male	376 (35.5)	1 (0.1)	375 (99.2)	
Transgender	4 (0.4)	4 (0.6)	0 (0)	
None of the above	9 (0.9)	6 (0.9)	3 (0.8)	
**Race (** ***n*** **, %)**				0.49
African American/Black	17 (1.6)	13 (1.9)	4 (1.1)	
Asian	78 (7.4)	46 (6.8)	32 (8.5)	
More than one race	53 (5.0)	31 (4.6)	22 (5.8)	
Other	49 (4.6)	34 (5.0)	15 (4.0)	
White	861 (81.4)	556 (81.8)	305 (80.7)	
**Ethnicity (** ***n*** **, %)**				0.5
Hispanic/Latinx	84 (7.9)	51 (7.5)	33 (8.7)	
Non-Hispanic/Latinx	974 (92.1)	629 (92.5)	345 (91.3)	
**Academic variables**			
**Credit hours** (*n*, %)				0.99
< 12 credits	123 (11.6)	79 (11.6)	44 (11.6)	
> 12 credits	935 (88.4)	601 (88.4)	334 (88.4)	
**Year in college** (*n*, %)				0.45
First	235 (22.2)	143 (21.0)	92 (24.3)	
Second	231 (21.8)	155 (22.8)	76 (20.1)	
Third	260 (24.6)	175 (25.7)	85 (22.5)	
Fourth	306 (28.9)	190 (27.9)	116 (30.7)	
Fifth or more	26 (2.5)	17 (2.5)	9 (2.4)	
**Residence type** (*n*, %)				0.41
On-campus/dormitories	314 (29.7)	196 (28.8)	118 (31.2)	
Not on-campus/dormitories	744 (70.3)	484 (71.2)	260 (68.8)	
**Job with in-person facing interaction** (*n*, %)				<0.001
No	760 (71.8)	461 (67.8)	299 (79.1)	
Yes	298 (28.2)	219 (32.2)	79 (20.9)	
**Health variables**			
**Perceived general health** (*n*, %)				<0.001
Excellent	378 (35.7)	210 (30.9)	168 (44.4)	
Very good	509 (48.1)	345 (50.7)	164 (43.4)	
Good	160 (15.1)	117 (17.2)	43 (11.4)	
Fair/poor	11 (1.0)	8 (1.2)	3 (0.1)	
**Depressive symptoms** (*n*, %)				<0.001
CES-D <10	616 (58.2)	350 (51.5)	266 (70.4)	
CES-D ≥10	442 (41.8)	330 (48.5)	112 (29.6)	
**Perceived stress symptoms** (*n*, %)				<0.001
Low	253 (23.9)	130 (19.1)	123 (32.5)	
Moderate	700 (66.2)	465 (68.4)	235 (62.2)	
High	105 (9.9)	85 (12.5)	20 (5.3)	
**Substance use variables**				
**Nicotine use (Past 30-days)** (*n*, %)				<0.001
No	729 (68.9)	187 (27.5)	14.2 (37.6)	
Yes	329 (31.1)	493 (72.5)	236 (62.4)	
**Drink alcohol** (*n*, %)				0.37
No	340 (32.1)	225 (33.1)	115 (30.4)	
Yes	718 (67.9)	455 (66.9)	263 (69.6)	
**COVID-19 variables**				
**Knew someone who died of SARS-CoV-2 infection** (n, %)				0.68
No	924 (87.3)	596 (87.6)	328 (86.8)	
Yes	134 (12.7)	84 (12.4)	50 (47.9)	
**Tested positive for SARS-CoV-2** (*n*, %)				0.48
No	947 (89.5)	612 (90.0)	335 (88.6)	
Yes	111 (10.5)	68 (10.0)	43 (11.4)	

**Table 2:** Characteristics of undergraduates by sleep duration.

**Characteristics**	**Total sample *n =* 1,058**	**Low (<7 h) *n =* 179**	**Recommended (7–9 h) *n =* 879**	***t*-test or chi-squared value**
Total %	100.0	16.9	83.1	
**Socio-demographic variables**			
**Age** (mean, sd)	19.96 (1.92)	20.16 (2.54)	19.92 (1.77)	1.18
**Sex at birth** (*n*, %)				0.25
Female	680 (64.3)	118 (17.4)	562 (82.6)	
Male	378 (35.7)	61 (16.1)	317 (83.4)	
**Gender** (*n*, %)				0.61
Female	669 (63.2)	116 (17.3)	553 (82.7)	
Male	376 (35.5)	61 (16.2)	315 (83.8)	
Transgender	4 (0.4)	1 (25.0)	3 (75.0)	
None of the above	9 (0.9)	1 (11.1)	8 (88.9)	
**Race** (*n*, %)				13.59^**^
African American/Black	17 (1.6)	7 (41.2)	10 (58.8)	
Asian	78 (7.4)	19 (24.4)	59 (75.6)	
More than one race	53 (5.0)	11 (20.8)	42 (79.2)	
Other	49 (4.6)	11 (22.4)	38 (77.6)	
White	861 (81.4)	131 (15.2)	730 (84.8)	
**Ethnicity** (*n*, %)				0.29
Hispanic/Latinx	84 (7.9)	16 (19.0)	68 (81.0)	
Non-Hispanic/Latinx	974 (92.1)	163 (16.7)	811 (83.3)	
**Sleep variables**				
**Sleep hours** (mean, sd)	7.29 (0.92)	5.78 (0.47)	7.60 (0.64)	
**My sleep was restless**. (*n*, %)				45.98^***^
Rarely or none of the time (<1 day)	404 (38.2)	44 (10.9)	360 (89.1)	
Some or a little of the time (1–2 days)	390 (36.9)	62 (15.6)	328 (84.1)	
Occasionally or a moderate amount of time (3–4 days)	201 (19.0)	46 (22.9)	155 (77.1)	
All of the time (5–7 days)	63 (6.0)	27 (42.9)	36 (57.1)	
**Academic variables**			
**Credit hours**				0.09
<12 credits	123 (11.6)	22 (12.3)	101 (11.5)	
>12 credits	935 (88.4)	157 (87.7)	778 (88.5)	
**Year in college** (*n*, %)				2.72
First	235 (22.2)	42 (17.9)	193 (82.1)	
Second	231 (21.8)	40 (17.3)	191 (82.7)	
Third	260 (24.6)	39 (15.0)	221 (85.0)	
Fourth	306 (28.9)	51 (16.7)	255 (83.3)	
Fifth or more	26 (2.5)	7 (26.9)	19 (73.1)	
**Residence type** (*n*, %)				0.76
On-campus/dormitories	314 (29.7)	58 (18.5)	256 (81.5)	
Not on-campus/dormitories	744 (70.3)	121 (16.3)	623 (83.7)	
**Job with in-person facing interaction** (*n*, %)				3.05
No	760 (71.8)	119 (15.7)	641 (84.3)	
Yes	298 (28.2)	60 (20.1)	238 (79.9)	
**Health variables**				
**Perceived general health**				14.60^***^
Excellent	378 (35.7)	56 (14.8)	322 (85.2)	
Very good	509 (48.1)	79 (15.5)	430 (84.5)	
Good	160 (15.1)	39 (24.4)	121 (75.6)	
Fair/poor	11 (1.0)	5 (45.5)	6 (54.5)	
**Depressive symptoms** (*n*, %)				4.13^*^
CES-D <10	616 (58.2)	92 (14.9)	524 (85.1)	
CES-D ≥10	442 (41.8)	87 (19.7)	355 (80.3)	
**Perceived stress symptoms** (*n*, %)				7.96^*^
Low	253 (23.9)	29 (11.5)	224 (88.5)	
Moderate	700 (66.2)	127 (18.5)	573 (81.9)	
High	105 (9.9)	23 (21.9)	82 (78.1)	
**Substance use variables**				
**Nicotine use (Past 30-days)** (*n*, %)				0.35
No	729 (68.9)	120 (16.5)	609 (83.5)	
Yes	329 (31.1)	59 (17.9)	270 (82.1)	
**Drink alcohol** (*n*, %)				0.37
No	340 (32.1)	61 (17.9)	279 (82.1)	
Yes	718 (67.9)	118 (16.4)	600 (83.6)	
**COVID-19 variables**				
**Knew someone who died of SARS-CoV-2 infection** (*n*, %)				6.48^*^
No	924 (87.3)	146 (15.8)	778 (84.2)	
Yes	134 (12.7)	33 (24.6)	101 (75.4)	
**Tested positive for SARS-CoV-2** (*n*, %)				6.85^**^
No	947 (89.5)	170 (18.0)	777 (82.0)	
Yes	111 (10.5)	9 (8.1)	102 (91.9)	

*Note*:

* * p <0.05*;

** * p <0.01*;

*** * p <0.001*.

Respondents who knew someone who had died from a SARS-CoV-2 infection were more likely to report a short sleep duration compared to those who did not know someone who had died from a SARS-CoV-2 infection (*x*^2^ = 6.48, *p* = <0.05), as presented in Table 2. However, respondents with a previous positive SARS-CoV-2 test result were less likely to report a short sleep duration compared to respondents without a previous positive SARS-CoV-2 test result (*x*^2^ = 6.85, *p* = <0.01).

### Outcome data

Our sample had a mean sleep duration of 7.29 h (SD = 0.92). One hundred seventy-nine (16.9%) respondents reported averaging less than the recommended sleep duration (less than 7 h), and most (*n* = 879; 83.1%) reported averaging the recommended seven to 9 h of sleep. The distribution of self-reported sleep duration is as follows: *n* = 4 averaged 4 h of sleep, *n* = 32 averaged 5 h of sleep, *n* = 143 averaged 6 h of sleep, *n* = 428 averaged 7 h of sleep, *n* = 378 averaged 8 h of sleep, and *n* = 73 averaged 9 h of sleep.

### Main results

#### Knew someone who died from a SARS-CoV-2 infection

In the unadjusted logistic regression model, respondents who knew someone who had died from a SARS-CoV-2 infection were nearly 75% more likely to report short sleep duration relative to respondents who did not know someone who had died from a SARS-CoV-2 infection (OR = 1.74, 95% CI: 1.12, 2.65). As presented in Table 3, after adjusting for the covariates, the results showed that respondents who knew someone who had died from a SARS-CoV-2 infection were more likely to report a short sleep duration, compared to respondents who did not know someone who had died from a SARS-CoV-2 infection (aOR = 1.80, 95% CI: 1.14, 2.79).

**Table 3:** Associations between knowing someone who died of SARS-CoV-2 infection and sleep duration, September 2020 (*n* = 1,058) Odds Ratio (95% CI).

**Variables**	**OR (95% CI)**	**aOR (95% CI)**
**Knew someone who died of**
**SARS-CoV-2 infection**
No	Ref	Ref
Yes	1.74 (1.12, 2.65)^*^	1.80 (1.14, 2.79)^*^

*Reference group for sleep duration was seven to 9 h*;

* *p <0.05*.

*Adjusted for age, sex, race, ethnicity, perceived general health, residence type, year in college, total credit hours, job with in-person interaction, nicotine use, alcohol, depressive symptoms, and perceived stress symptoms*.

#### Tested positive for SARS-CoV-2 infection

As shown in Table 4, participants with a positive SARS-CoV-2 test history were less likely to report sleeping short sleep duration when compared to those who without a previous positive test result for a SARS-CoV-2 infection (OR = 0.40, 95% CI: 0.19, 0.77). Similarly, after adjusting for the covariates, those who reported having tested positive for SARS-CoV-2 infection were less likely to sleep a short duration, compared to respondents who reported not having a history of testing positive for a SARS-CoV-2 infection (aOR = 0.47, 95% CI: 0.21, 0.91).

**Table 4:** Associations between testing positive for SARS-CoV-2 and sleep duration, september 2020 (*n* = 1,058) Odds Ratio (95% CI).

**Variables**	**OR (95% CI)**	**aOR (95% CI)**
**Tested Positive for SARS-CoV-2**
No	Ref	Ref
Yes	0.40 (0.19, 0.77)^*^	0.47 (0.21, 0.91)^*^

*Reference group for sleep duration was seven to 9 h*;

* *p <0.05*.

*Adjusted for age, sex, race, ethnicity, perceived general health, residence type, year in college, total credit hours, job with in-person interaction, nicotine use, alcohol, depressive symptoms, and perceived stress symptoms*.

## Discussion

### Main finding of this study

In this study of college students conducted during the Fall 2020 semester, knowing someone who died from a SARS-CoV-2 infection and having tested positive for SARS-CoV-2 both had significant associations with sleep duration, but in opposite directions. Specifically, students who knew someone who had died from a SARS-CoV-2 infection were less likely to report averaging the recommended seven to 9 h of sleep compared to the students who did not know someone who had died from a SARS-CoV-2 infection. However, students who had received a positive SARS-CoV-2 test result were more likely to sleep the recommended seven to 9 h per night, when compared to students without a positive test result history.

An Arizona longitudinal COVID-19 study known as CoVHORT that assessed the differences in sleep duration between laboratory-confirmed SARS-CoV-2 positive vs. negative status among adults with an average age of approximately 47 found sleep duration overall had increased during the pandemic (33). Interestingly, although sleep duration overall increased, Arizona residents who were positive for SARS-CoV-2 reported longer sleep durations compared to those who had tested negative (33).

Undergraduate students are a generally young and healthy population who have unique stressors that make them more vulnerable to insufficient sleep. This study offers an insight into the association between sleep duration and two SARS-CoV-2 experiences, knowing someone who had died of SARS-CoV-2 infection and having received a positive SARS-CoV-2 test result, on a younger and healthier subpopulation while taking into account undergraduate student's unique stressors of enrolled credit hours, living on campus, depressive symptoms, and perceived stress symptoms. The associations between students' prior SARS-CoV-2 infection status and knowing someone who died from a SARS-CoV-2 infection and sleep duration identified in the current study highlights the importance of gaining an understanding on how personal life events caused by the pandemic or other unusual major events might be associated with students' sleep health. This association between factors related to the COVID-19 pandemic and sleep duration shows additional research is needed to better understand the pandemic's impact beyond the earlier days of the lockdown. The long-lasting effects of grief have been reported during the aftermath of other infectious disease outbreaks (21). In this sample of college students, those who knew someone who had died as a result of COVID-19 were more likely to report a short sleep duration. Supporting students who have experienced a loss because of a SARS-CoV-2 infection could therefore help improve student's sleep duration and overall health.

Our finding that students who received a positive SARS-CoV-2 test result were less likely to report short sleep duration is in line with the findings from the study of Arizona adults described above. For those who tested positive for SARS-CoV-2, some may have been asymptomatic, in which they had no symptoms and felt no change in their health. Among those that did experience symptoms, the severity experienced by the respondents could have been quite mild, such as a cough, fever, chills, fatigue, headache, sore throat, or loss of taste and/or smell. Severe symptoms could have included difficulty breathing or inability to stay awake, that would have required hospitalization and possibly being intubated to be connected to a ventilator to assist with breathing. Unfortunately, symptom severity is unknown for the respondents in our study sample. These differences in symptomology would have resulted in varying sleep duration among the students who tested positive for SARS-CoV-2. Differences in sleep duration based on SARS-CoV-2 testing history might be explained by the fatigue syndrome experienced by recovered COVID-19 patients (34), or it could be a consequence of rumination or fear of contagion experienced by students who had not been infected (19). In other words, students who had tested positive but did not suffer severe consequences from their SARS-CoV-2 infection did not have the fear of the unknown as a potential factor to disrupt their sleep.

### Limitations

Our study has a few limitations. First, participants reported their average sleep duration, so more details regarding sleep (i.e., differences in weekday vs. weekend sleep, daytime/nap data, time it usually took people to fall asleep each night, and whether they woke up in the middle of the night or early morning) are unknown for this sample. Second, this study assessed a single component of sleep health (i.e., sleep duration), which does not account for all the dimensions of sleep. However, sleep duration is a valid measurement that has been previously used as the main outcome variable by recent studies focused on sleep and its association with health factors (25, 35, 36). The third limitation stems from the cross-sectional study design. As an observational study we are unable to determine directionality or causality between the two SARS-CoV-2 experiences, knowing someone who died from SARS-CoV-2 infection and having a positive SARS-CoV-2 test result and sleep duration. It may be possible that respondents who slept more were more susceptible to a SARS-CoV-2 infection and that the respondents who know someone who died from a SARS-CoV-2 infection may have been already sleeping less hours prior to the event. The fourth limitation to mention is the inclusion of self-reported information, specifically the SARS-CoV-2 test result. However, for the self-reported SARS-CoV-2 test results it is important to note that students were regularly tested by the university. Furthermore, during fall 2020 people were vigilant about their SARS-CoV-2 infection status as COVID-19 disease was spreading around the world. Therefore, it would have been very unlikely for a student to misremember a positive SARS-CoV-2 test result administered by the university. Finally, there were too few participants who had self-reported long (10+ h) sleep duration, therefore we could not test for differences between the long sleep duration and recommended sleep groups because it would introduce small sampling bias. Similar to Lu et al.'s (25) study using the NSF's sleep recommendation guidelines, we restricted the sample and made the comparison between only short and the recommended sleep durations.

## Conclusions

This study's findings suggest that college students' sleep might be associated with their personal experience with both knowing someone who had died of SARS-CoV-2 infection and having received a positive SARS-CoV-2 test result. However, different experiences may impact sleep differently, so further research is warranted to better understand how unusual events, like unexpected pandemics, impact the sleep of college students. In particular, longitudinal investigation into the relationship between such experiences and sleep is needed to establish causal relationships. Regardless, sleep hygiene interventions in higher education should consider taking into account the impact the COVID-19 pandemic has had and may continue to have on students' sleep.

## Data availability statement

The raw data supporting the conclusions of this article will be made available by the authors, without undue reservation.

## Ethics statement

The studies involving human participants were reviewed and approved by the Indiana University Human Subjects and Institutional Review Boards. The participants provided their written informed consent to participate in this study.

## Author contributions

EG: conceptualization, methodology, software, validation, formal analysis, investigation, data curation, writing-original draft, and visualization. CC: conceptualization, investigation, data curation, and writing-review & editing. SK: validation, investigation, data curation, and writing-review & editing. CL and MR: supervision, conceptualization, methodology, formal analysis, investigation, resources, writing-review & editing, project administration, and funding acquisition. JM: supervision, conceptualization, methodology, formal analysis, investigation, resources, writing-review & editing, visualization, project administration, and funding acquisition. All authors contributed to the article and approved the submitted version.

## Funding

This work was supported by a private donation to the Indiana University Foundation.

## Conflict of interest

The authors declare that the research was conducted in the absence of any commercial or financial relationships that could be construed as a potential conflict of interest.

## Publisher's note

All claims expressed in this article are solely those of the authors and do not necessarily represent those of their affiliated organizations, or those of the publisher, the editors and the reviewers. Any product that may be evaluated in this article, or claim that may be made by its manufacturer, is not guaranteed or endorsed by the publisher.
