# Supplementary material for: The relationship between acquaintance with a SARS-CoV-2 death, positive SARS-CoV-2 test results, and sleep duration among college students in fall 2020
Source: Front Public Health. 2022 Aug 18;10:949438. doi: 10.3389/fpubh.2022.949438 (PMC9434826; doi:10.3389/fpubh.2022.949438)
Supplement: Supplementary file 1 [file Table_1.DOCX]

**Appendix**

***Demographics***

1. **Age.** *What is your current age?* Numeric
2. **Sex**. *What sex were you assigned at birth, on your original birth certificate?*

1 = male (reference)

2 = female.

1. **Race and ethnicity.** *Which categories describe you? (Please choose all that apply)*

1 = White

2 = Hispanic or Latinx or Spanish origin

3 = Black or African American

4 = Asian

5 = American Indian or Alaska Native

6 = Middle Eastern or North African

7 = Native Hawaiian or Other Pacific Islander

8 = Some other race, ethnicity, or origin.

1. **Perceived General Health.** “*In general, how would you describe your health?*” with options

1 = excellent

2 = very good

3 = good

4 = fair

5 = poor.

1. **Residence Type.** Options for residence type included,

1 = On campus (in a dorm/residence hall)

2 = Off campus apartment

3 = Off campus house

4 = At home with parents or other family members

5 = Other

***Academic***

1. **Year in college.** *What is your year in school?*

1 = First year undergraduate

2 = Second year undergraduate

3 = Third year undergraduate

4 = Fourth year undergraduate

5 = Fifth or more year undergraduate

1. **Total credit hours.** *How many credit hours are you taking this semester?* Numeric 0 – 30.
2. **Job with in-person interaction.** *Do you have a job or internship that requires you to* *interact face-to-face (in-person/non-virtually) with others?*

0 = No (reference)

1 = Yes.

***Substance use***

1. **Past 30-day Nicotine Use.** *During the last 30 days, on how many days did you use e-cigarettes?*

1 = Cigarettes

2 = E-cigarettes

3 = Other inhaled products

4 = None of the above

1. **Alcohol.** *How many days a week do you usually drink alcohol?*

0 = 0 days or don’t drink alcohol

1 = 1 day

2 = 2 days

3 = 3 days

4 = 4 days

5 = 5 days

6 = 6 days

7 = 7 days

***Mental Health***

1. **Depressive Symptoms (CES-D 10)** [1, 2].

0 = Rarely or none of the time (less than 1 day); 1 = Some or a little of the time (1-2 days); 2 = Occasionally or a moderate amount of time (3-4 days); 3 = All of the time (5-7 days)

- 1. I was bothered by things that usually don't bother me.
  2. I had trouble keeping my mind on what I was doing.
  3. I felt depressed.
  4. I felt that everything I did was an effort.
  5. I felt hopeful about the future.*
  6. I felt fearful.
  7. My sleep was restless.
  8. I was happy.*
  9. I felt lonely.
  10. I could not "get going."

1. **Perceived Stress Symptoms (PSS-10)** [3].

0 = Never; 1 = Almost Never; 2 = Sometimes; 3 = Fairly Often; 4; Very Often

- 1. In the last month, how often have you been upset because of something that happened unexpectedly?
  2. In the last month, how often have you felt that you were unable to control the important things in your life?
  3. In the last month, how often have you felt nervous and "stressed"?
  4. In the last month, how often have you felt confident about your ability to handle your personal problems?*
  5. In the last month, how often have you felt that things were going your way?*
  6. In the last month, how often have you found that you could not cope with all the things that you had to do?
  7. In the last month, how often have you been able to control irritations in your life?*
  8. In the last month, how often have you felt that you were on top of things?*
  9. In the last month, how often have you been angered because of things that were outside of your control?
  10. In the last month, how often have you felt difficulties were piling up so high that you could not overcome them?

*reverse coded

**References**

1. Andresen EM, Malmgren JA, Carter WB, Patrick DL. Screening for depression in well older adults: Evaluation of a short form of the CES-D. Am. J. Prev. Med. 1994; 10(2):77-84. doi:10.1016/s0749-3797(18)30622-6

2. Radloff LS. The CES-D Scale: A Self-Report Depression Scale for Research in the General Population. Appl. Psychol. Meas. 1977; 1(3):385-401. doi:10.1177/014662167700100306

3. Cohen S, Kamarck T, Mermelstein R. A Global Measure of Perceived Stress. J. Health Soc. Behav. 1983; 24(4):385-96. doi:<https://doi.org/10.2307/2136404>
